# EPEC Recruits a Cdc42-Specific GEF, Frabin, To Facilitate PAK Activation and Host Cell Colonization

**DOI:** 10.1128/mBio.01423-20

**Published:** 2020-11-03

**Authors:** Vikash Singh, Peter J. Hume, Anthony Davidson, Vassilis Koronakis

**Affiliations:** a Department of Pathology, University of Cambridge, Cambridge, United Kingdom; Washington University School of Medicine; Washington University School of Medicine

**Keywords:** EPEC, EspG, GTPases, actin

## Abstract

Enteropathogenic Escherichia coli (EPEC) is an extracellular pathogen that tightly adheres to host cells by forming “actin pedestals” beneath the bacteria, a critical step in pathogenesis. EPEC injects effector proteins that manipulate host cell signaling cascades to trigger pedestal assembly. We have recently shown that one such effector, EspG, hijacks p21-activated kinase (PAK) and sustains its activated state to drive the cytoskeletal changes necessary for attachment of the pathogen to target cells. This EspG subversion of PAK required active Rho family small GTPases in the host cell. Here we show that EPEC itself promotes the activation of Rho GTPases by recruiting Frabin, a host guanine nucleotide exchange factor (GEF) for the Rho GTPase Cdc42. Cells devoid of Frabin showed significantly lower EPEC-induced PAK activation, pedestal formation, and bacterial attachment. Frabin recruitment to sites of EPEC attachment was driven by EspG and required localized enrichment of phosphatidylinositol 4,5-bisphosphate (PIP2) and host Arf6. Our findings identify Frabin as a key target for EPEC to ensure the activation status of cellular GTPases required for actin pedestal formation.

## Abstract (precis)

Enteropathogenic Escherichia coli (EPEC) is a leading cause of diarrhea in children, especially in the developing world. EPEC initiates infection by attaching to cells in the host intestine, triggering the formation of actin-rich “pedestal” structures directly beneath the adherent pathogen. These bacteria inject their own receptor into host cells, which upon binding to a protein on the pathogen surface triggers pedestal formation. Multiple other proteins are also delivered into the cells of the host intestine, which work together to hijack host signaling pathways to drive pedestal production. Here we show how EPEC hijacks a host protein, Frabin, which creates the conditions in the cell necessary for the pathogen to manipulate a specific pathway that promotes pedestal formation. This provides new insights into this essential early stage in disease caused by EPEC.

## INTRODUCTION

Enteropathogenic Escherichia coli (EPEC) and enterohemorrhagic E. coli (EHEC) are bacterial pathogens that are responsible for significant morbidity and mortality globally (1, 2). EPEC causes diarrhea in children, especially in the developing world (3), while EHEC is associated with outbreaks of acute gastroenteritis and bloody diarrhea, often linked to contaminated food and sometimes leading to life-threatening complications (4). Following ingestion, EPEC and EHEC tightly adhere to intestinal epithelial cells and cause morphological changes leading to the loss of brush border microvilli, forming characteristic attaching and effacing (A/E) lesions (5). Extensive reorganization of the host cell cytoskeleton beneath the adherent pathogens leads to the formation of characteristic actin “pedestals” (6). These structures, which are seemingly crucial for pathogenesis (6), strengthen the attachment of the bacteria to the host epithelium, and also may drive the movement of the adherent pathogen across the epithelial surface, promoting the formation of microcolonies and potentially allowing spread to adjacent cells (7, 8).

Both EPEC and EHEC utilize a type 3 secretion system (T3SS) to deliver a battery of virulence effector proteins into host cells in order to subvert the cellular signaling networks necessary to drive the cytoskeletal rearrangements underlying pedestal formation (9). We recently reported that one of these effectors, EspG, hijacks host p21-activated kinase (PAK) to facilitate pedestal formation and bacterial attachment (10). EspG was able to subvert PAK only in the presence of active Rho family small GTPases, which function to both concentrate PAK at the membrane and stimulate PAK activation (10).

Rho GTPases are master regulators of numerous eukaryotic signaling networks (11) and are consequently common targets for subversion by bacterial pathogens (12). For example, several bacteria release toxins that modify GTPases to bring about their permanent activation (13). Other pathogens deliver effectors that mimic host regulators of GTPase signaling, such as guanine nucleotide exchange factors (GEFs; activators) (14) and GTPase-activating proteins (GAPs; inactivators) (15). EPEC and EHEC are themselves known to encode several effectors that have GEF activity (16–18). However, whether these contribute to the GTPase activation required for EspG-dependent hijacking of PAK or whether some other host or pathogen factor is responsible is unknown. Here, we sought to determine how EPEC ensures a sufficient level of Rho GTPase activation in target host cells.

## RESULTS

### EPEC activates Rho GTPases.

**FIG 1:**
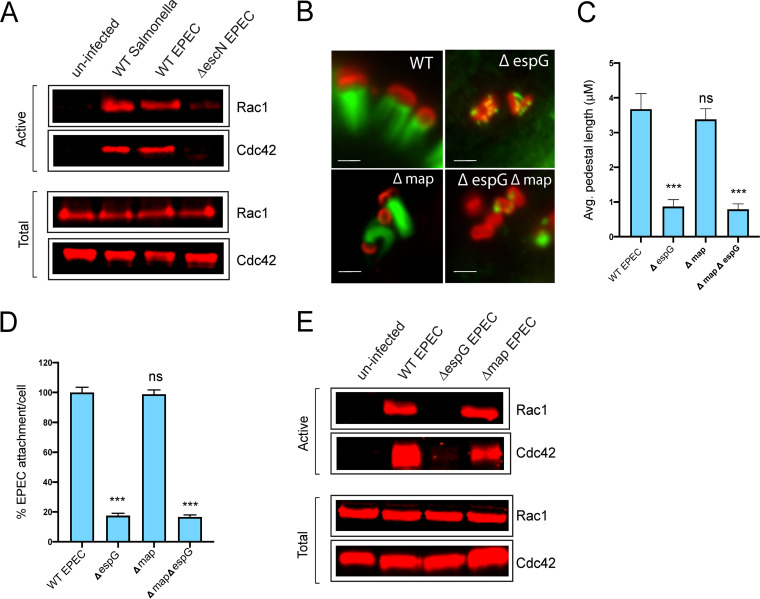
EPEC activates Cdc42 upon attachment to host cells. (A) Immunoblot of the level of Cdc42 and Rac1 isolated by GST-GBD beads (“active”) from lysates prepared from either uninfected Hap1 cells or those infected with WT *Salmonella*, WT EPEC, or Δ*escN* EPEC, as indicated. Also shown is the total level of Cdc42 and Rac1 (active and inactive) in the lysates (“total”). (B) Fluorescence microscopy images of actin pedestals formed on Hap1 cells by WT, Δ*espG*, Δ*map*, and Δ*espG* Δ*map* EPEC bacteria at 60 min postinfection. Actin (green) is stained with Alexa Fluor 488-phalloidin, and bacteria (red) are stained with anti-intimin antibody. Scale bar, 1 μm. (C) Average pedestal length produced by EPEC strains as described above for panel B. Each bar represents the average of results from three separate experiments (100 to 200 cells for each replicate, 300 to 500 cells in total). (D) Quantification of the attachment of strains described above for panel B to WT Hap1 cells relative to that of WT EPEC (there are typically 6 or 7 WT EPEC bacteria per cell). Error bars indicate standard deviations (SD). ***, *P* < 0.001; ns, not significant (by one-way analysis of variance [ANOVA] followed by a *post hoc* Dunnett comparison) relative to WT EPEC attachment. (E) Immunoblot of the level of Cdc42 and Rac1 isolated by GST-GBD beads (“active”) from lysates prepared from either uninfected Hap1 cells, or those infected with WT EPEC, Δ*espG* EPEC or Δ*map* EPEC, as indicated. Also shown is the total level of Cdc42 and Rac1 in the lysates (“total”).

To determine the source of the active Rho GTPases required for EPEC to hijack host PAK (10), we first sought to determine whether the level of active GTPases changes in response to infection. EPEC-infected cell lysates were incubated with GST-PAK-PBD (GST stands for glutathione *S*-transferase, and PBD stands for protein-binding domain) beads, which specifically interact with the active form of Rac1 and Cdc42. EPEC infection resulted in significant GTPase activation, similar to that seen upon control infection with *Salmonella* (Fig. 1A; see also Fig. S1A in the supplemental material). In contrast, infection with Δ*escN* EPEC, which lack a functional type 3 secretion system, triggered negligible GTPase activation, suggesting that an EPEC-delivered effector is responsible for triggering activation.

10.1128/mBio.01423-20.1 FIG S1 (A) Quantification of the level of Cdc42 isolated by GST-GBD beads (“active”) from lysates prepared from either uninfected Hap1 cells or those infected with WT *Salmonella*, WT EPEC, or Δ*escN* EPEC as indicated. The levels are quantified from Western blot images shown in Fig. 1A. Each bar represents the average from three separate experiments; error bars represent standard deviations (SD). ***, *P* < 0.001; NS, not significant (one-way ANOVA followed by a *post hoc* Dunnett comparison) relative to control uninfected cells. (B) Quantification of the level of Cdc42 isolated by GST-GBD beads (“active”) from lysates prepared from either uninfected Hap1 cells or those infected with WT EPEC, Δ*espG* EPEC, or Δ*map* EPEC as indicated. The levels are quantified from Western blot images shown in Fig. 1E. Each bar represents the average from three separate experiments; error bars represent SD. ***, *P* < 0.001; NS, not significant (one-way ANOVA followed by a *post hoc* Dunnett comparison) relative to control uninfected cells. (C) (i) Immunoblot of the level of Cdc42 isolated by GST-GBD beads (“active”) from lysates prepared from either uninfected LoVo cells or those infected with WT EPEC, Δ*espG* EPEC, Δ*map* EPEC, or Δ*espG* Δ*map* EPEC as indicated. Also shown is the total level of Cdc42 in the lysates (“total”). (ii) Quantification of the Western blots shown in (ii). Each bar represents the average from three separate experiments; error bars represent SD. ***, *P* < 0.001; **, *P* < 0.01; NS, not significant (one-way ANOVA followed by a *post hoc* Dunnett comparison) relative to control uninfected cells. (D) (i) Immunoblot of the level of Cdc42 isolated by GST-GBD beads (“active”) from lysates prepared from either uninfected HeLa cells or those infected with WT EPEC, Δ*espG* EPEC, Δ*map* EPEC, or Δ*espG* Δ*map* EPEC as indicated. Also shown is the total level of Cdc42 in the lysates (“total”). (ii) Quantification of the Western blots shown in panel i. Each bar represents the average from three separate experiments; error bars represent SD. ***, *P* < 0.001; **, *P* < 0.01; NS, not significant (one-way ANOVA followed by a *post hoc* Dunnett comparison) relative to control uninfected cells. (E) (i) Immunoblot of the level of Cdc42 isolated by GST-GBD beads (“active”) from lysates prepared from either uninfected Caco-2 cells or those infected with WT EPEC, Δ*espG* EPEC, Δ*map* EPEC, or Δ*espG*Δ*map* EPEC as indicated. Also shown is the total level of Cdc42 in the lysates (“total”). (ii) Quantification of the Western blots shown in panel ii. Each bar represents the average from three separate experiments; error bars represent SD. ***, *P* < 0.001; **, *P* < 0.01; NS, not significant (one-way ANOVA followed by a *post hoc* Dunnett comparison) relative to control uninfected cells. Download FIG S1, TIF file, 0.6 MB. Copyright © 2020 Singh et al. 2020 Singh et al. This content is distributed under the terms of the Creative Commons Attribution 4.0 International license.

The EPEC effector Map (mitochondrion-associated protein) is known to be a GEF for Cdc42, and indeed, it has been shown to trigger Cdc42-dependent filopodium formation upon EPEC infection (19, 20). However, unlike Δ*espG* EPEC, Δ*map* EPEC showed no defect in either pedestal formation (Fig. 1B and C) or attachment to cultured Hap1 cells (Fig. 1D). A double Δ*espG* Δ*map* strain displayed no additional defect in either phenotype compared to Δ*espG* EPEC, suggesting that Map is not required to activate the GTPases necessary for the hijacking of PAK and consequent pedestal formation. Indeed, in our cell culture infection model, Δ*map* EPEC activated Rho GTPases to a level equivalent to that of wild-type (WT) EPEC (ig. 1E, Fig. S1B). However, to our surprise, control Δ*espG* EPEC was unable to induce GTPase activation. Similar results were observed in multiple cell lines (Fig. S1C to E). In some cell types (Caco-2 and LoVo), the level of Cdc42 activation was lower in cells infected with Δ*map* EPEC than those infected with WT EPEC, suggesting that Map does play a role in Cdc42 activation. However, in all cases, this reduction was much smaller than that seen in cells infected with either Δ*espG* EPEC or the Δ*espG* Δ*map* strain in which Cdc42 activation was abolished. As EspG has no structural or sequence similarity to known GEFs and has never been reported to possess GEF activity, this suggests that EPEC may exploit a host cell GEF to induce the GTPase activity required for EspG-mediated hijacking of PAK and that seemingly EspG itself plays a key role in this process.

### EPEC recruits a host Cdc42 GEF to induce GTPase activation.

**FIG 2:**
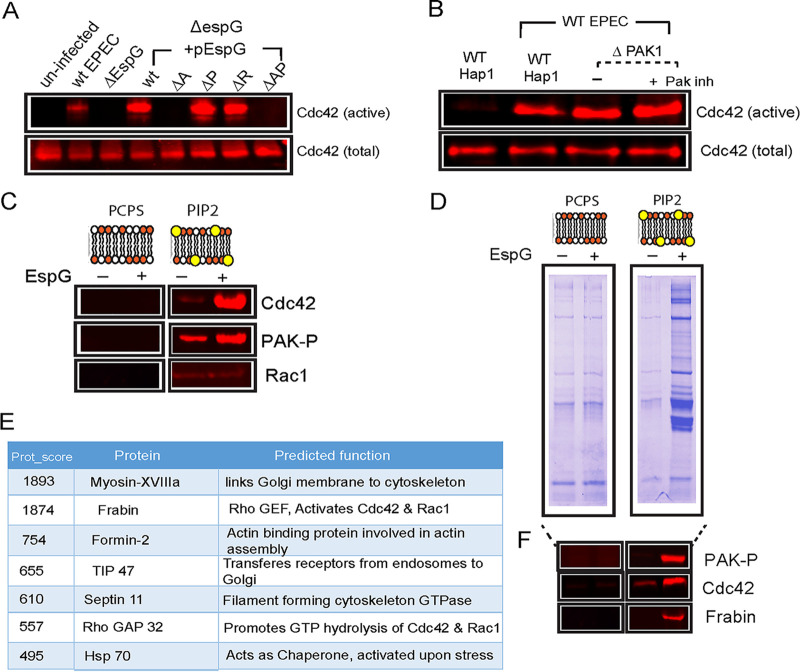
EPEC recruits the Cdc42 GEF Frabin. (A) Immunoblot of the level of Cdc42 isolated by GST-GBD beads (“active”) from lysates prepared from Hap1 cells infected with the indicated EPEC strains. Also shown is the total level of Cdc42 in the lysates (“total”). (B) Immunoblot of the level of Cdc42 isolated by GST-GBD beads (“active”) from lysates prepared from either uninfected WT Hap1 cells or EPEC-infected WT HAP1, ΔPAK1 Hap1, or ΔPAK1 cells pretreated with PAK inhibitor as indicated. (C) Immunoblot showing recruitment of the indicated proteins from porcine brain extract by PC:PI or PC:PI:PIP2 (“PIP2”) lipid bilayers in the presence (+) or absence (−) of EspG. (D) SDS-PAGE analysis and Coomassie blue staining of proteins recruited by PC:PI and PC:PI:PIP2 (“PIP2”) lipid bilayers from porcine brain extract in the presence and absence of EspG. (E) Top hits from mass spectrometry analysis of specific proteins recruited by PC:PI:PIP2 bilayers in the presence of EspG. (F) Immunoblot showing the levels of indicated proteins in samples from panel D.

PAK function in cells requires Rho GTPases (21), but PAK can also itself promote activation of GTPases, for example via PIX (PAK-interacting exchange factor) (22), leading to amplification of PAK signaling. It is therefore possible that the EspG-dependent increase in active Rho GTPases upon EPEC infection is due to EspG hijacking the PAK cascade. To test this possibility, we performed a GTPase activation assay in HAP1 cells infected with Δ*espG* EPEC complemented with various derivatives of EspG (Fig. 2A, Fig. S2A). Complementation with WT EspG restored the ability of Δ*espG* EPEC to activate Cdc42, whereas derivatives deficient in binding to Arf GTPases alone or to both Arf and PAK were unable to restore Rho activation. This is presumably because the interaction with Arf GTPases is required for correct localization of EspG. Mutations that disrupt the binding of EspG to either Rab GTPases (ΔR) or PAK (ΔP) did not affect Rho GTPase activation, suggesting that EspG can signal to Rho proteins independently of PAK, and therefore also PIX. In confirmation of this, Cdc42 was efficiently activated by EPEC in either ΔPAK1 cells, or ΔPAK1 cells treated with a chemical inhibitor of other class I PAK isoforms (FRAX486) (Fig. 2B, Fig. S2B).

10.1128/mBio.01423-20.2 FIG S2 (A) Quantification of the level of Cdc42 isolated by GST-GBD beads (“active”) from lysates prepared from Hap1 cells infected with the indicated EPEC strains. The levels are quantified from Western blot images shown in Fig. 2A. Each bar represents the average from three separate experiments; error bars represent SD. ***, *P* < 0.001; NS, not significant (one-way ANOVA followed by a *post hoc* Dunnett comparison) relative to control uninfected cells. (B) Quantification of the level of Cdc42 isolated by GST-GBD beads (“active”) from lysates prepared from either uninfected WT Hap1 cells or EPEC-infected WT HAP1, ΔPAK1 Hap1, or ΔPAK1 cells pretreated with PAK inhibitor as indicated. The levels are quantified from Western blot images shown in Fig. 2B. Each bar represents the average from three separate experiments; error bars represent SD. ***, *P* < 0.001 (one-way ANOVA followed by a *post hoc* Dunnett comparison) relative to control uninfected cells. Download FIG S2, TIF file, 0.2 MB. Copyright © 2020 Singh et al. 2020 Singh et al. This content is distributed under the terms of the Creative Commons Attribution 4.0 International license.

In order to identify the signaling network that leads to GTPase activation, we attempted to reconstitute EspG signaling *in vitro* using lipid bilayer-coated silica microspheres incubated in cell-free porcine brain extract. As we have previously shown, EspG anchored to microspheres coated with PC:PS (phosphatidylcholine:phosphatidylserine) bilayers fails to recruit any PAK, and it also fails to recruit any endogenous Cdc42 (Fig. 2C). Lipid bilayers designed to further mimic the site of action of EspG by including PIP2 (phosphatidylinositol 4,5-bisphosphate), known to be enriched at sites of EPEC attachment to host cells (23), recruited active, phosphorylated PAK (PAK-P) and a small amount of Cdc42. This was significantly enhanced by the presence of EspG, which leads to efficient recruitment of both Cdc42 and phosphorylated PAK (Fig. 2C). This confirms that EspG can trigger recruitment and activation of Cdc42 and suggests that membrane lipid composition is of critical importance in this EspG-mediated signaling to GTPases.

To attempt to identify how EspG signals to Rho GTPases, the proteins recruited from porcine brain extracts to PC:PS:PIP2-coated microspheres in the presence and absence of anchored EspG were separated by sodium dodecyl sulfate-polyacrylamide gel electrophoresis (SDS-PAGE) (Fig. 2D) and identified by parallel mass spectrometry (see Table S1 in the supplemental material). The top hits are summarized in Fig. 2E. Of particular note, one of the proteins enriched in the presence of EspG was a Cdc42-specific GEF, Frabin (also known as FGD4, FYVE, RhoGEF, and PH Domain Containing 4) (24, 25). The recruitment of Frabin was further confirmed using immunoblotting (Fig. 2F).

10.1128/mBio.01423-20.5 TABLE S1 Proteins recruited from porcine brain extracts to PC:PS:PIP2-coated microspheres in the presence and absence of anchored EspG were separated by SDS-PAGE and identified by parallel mass spectrometry. Download Table S1, XLS file, 0.1 MB. Copyright © 2020 Singh et al. 2020 Singh et al. This content is distributed under the terms of the Creative Commons Attribution 4.0 International license.

**FIG 3:**
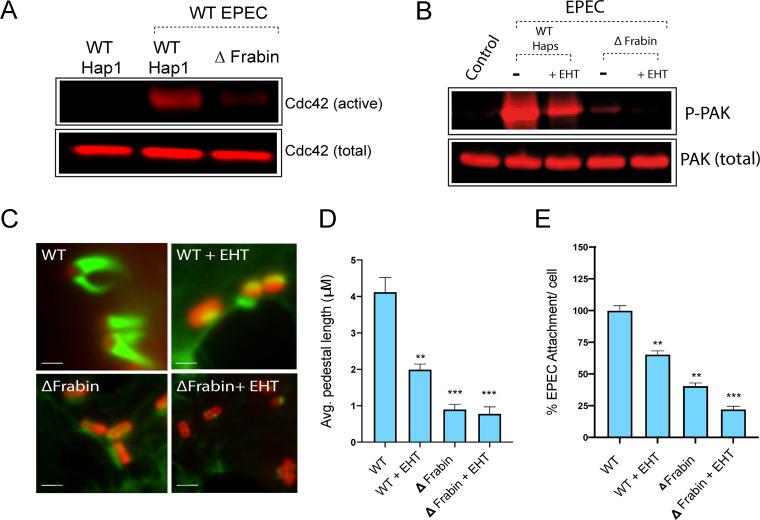
Frabin is important for EPEC pedestal and attachment. (A) Immunoblot of the level of Cdc42 isolated by GST-GBD beads (“active”) from lysates prepared from either uninfected WT Hap1 or EPEC-infected WT or ΔFrabin Hap1 cells. Also shown is the total level of Cdc42 in the lysates (“total”). (B) Immunoblot of the level of total (PAK) and active (phosphorylated on serine 144; PAK-P) PAK in uninfected WT Hap1 cells and EPEC-infected WT or ΔFrabin cells, with and without pretreatment with Rac inhibitor EHT 1864. (C) Fluorescence microscopy images of actin pedestals formed by WT EPEC on WT or ΔFrabin HAP1 cells, with and without pretreatment with Rac inhibitor EHT 1864. Actin (green) is stained with Alexa Fluor 488-phalloidin, and bacteria (red) are stained with anti-intimin antibody. Scale bar, 1 μm. (D) Average pedestal length formed by WT EPEC on cells described above for panel C. (E) Quantification of the attachment of EPEC to cells treated as in panel C, relative to that of WT EPEC attachment on WT HAP1 cells (there are typically 6 or 7 WT EPEC bacteria per WT HAP1 cell). Each bar represents the average of results from three separate experiments (100 to 200 cells for each replicate, 300 to 500 cells in total). Error bars indicate SD. ***, *P* < 0.001; **, *P* < 0.05; ns, not significant (by one-way analysis of variance [ANOVA] followed by a *post hoc* Dunnett comparison) relative to attachment on WT HAP1 cells.

We next examined whether Frabin plays a role in EPEC attachment and pedestal formation. In contrast to WT Hap1 cells, when Frabin knockout Hap1 cells (ΔFrabin) were infected with WT EPEC, virtually no increase was seen in the level of active Cdc42 (Fig. 3A, Fig. S3A). The level of active, phosphorylated PAK induced by EPEC infection was also much lower in ΔFrabin cells than in WT cells (Fig. 3B, Fig. S3B). There was still a residual level of PAK phosphorylation triggered in ΔFrabin cells; as Frabin is a Cdc42-specific GEF, this may be due to Rac1 activation, triggered by a separate pathway. Consistent with this, treatment of ΔFrabin cells with a Rac1 inhibitor (EHT1864) completely abolished EPEC-induced phosphorylation of PAK, whereas PAK phosphorylation was only partially reduced in WT Hap1 cells (Fig. 3B, Fig. S3B). Pedestals formed by WT EPEC on ΔFrabin cells were less efficient in terms of actin recruitment and had a different morphology compared to those formed on control HAP1 cells (Fig. 3C). Both pedestal length (Fig. 3D) and bacterial attachment (Fig. 3E) were significantly impaired by either knockdown or knockout of Frabin. An equivalent reduction in EPEC attachment was observed in HeLa, Caco-2, and LoVo cells treated with Frabin small interfering RNA (siRNA) (Fig. S3C and D). As seen for PAK phosphorylation above, pedestal length and bacterial attachment showed a small further decrease upon inhibition of Rac1 in ΔFrabin cells and only a partial reduction in WT Hap1 cells (Fig. 3D and E). Collectively, these data show that EPEC generates the level of active Cdc42 in infected cells required for subsequent hijacking of PAK via the host GEF Frabin.

10.1128/mBio.01423-20.3 FIG S3 (A) Quantification of the level of Cdc42 isolated by GST-GBD beads (“active”) from lysates prepared from either uninfected WT Hap1, or EPEC-infected WT or ΔFrabin Hap1 cells. The levels are quantified from Western blot images shown in Fig. 3A. Each bar represents the average from three separate experiments; error bars represent SD. ***, *P* < 0.001; *, *P* < 0.1 (one-way ANOVA followed by a *post hoc* Dunnett comparison) relative to control uninfected cells. (B) Quantification of the level of active (phosphorylated on serine 144; PAK-P) PAK in uninfected WT Hap1 cells and EPEC-infected WT or ΔFrabin cells, with and without pretreatment with Rac inhibitor EHT 1864. The levels are quantified from Western blot images shown in Fig. 3B and normalized by the level of total PAK. Each bar represents the average from three separate experiments; error bars represent SD. ***, *P* < 0.001; *, *P* < 0.1 (one-way ANOVA followed by a *post hoc* Dunnett comparison) relative to control uninfected cells. (C)Quantification of the attachment of EPEC to HeLa, LoVo, or Caco-2 cells (as indicated) treated with Frabin siRNA relative to untreated cells. Each bar represents the average of results from three separate experiments (100 to 200 cells for each replicate, 300 to 500 cells in total). Error bars indicate SD. **, *P* < 0.01 (by one-way analysis of variance [ANOVA] followed by a *post hoc* Dunnett comparison). (D) Western blot showing the level of Frabin in cells treated with Frabin siRNA as described above for panel C. Download FIG S3, TIF file, 0.3 MB. Copyright © 2020 Singh et al. 2020 Singh et al. This content is distributed under the terms of the Creative Commons Attribution 4.0 International license.

### The DH and PH domains of Frabin are essential for Rho GTPase activation by EPEC.

**FIG 4:**
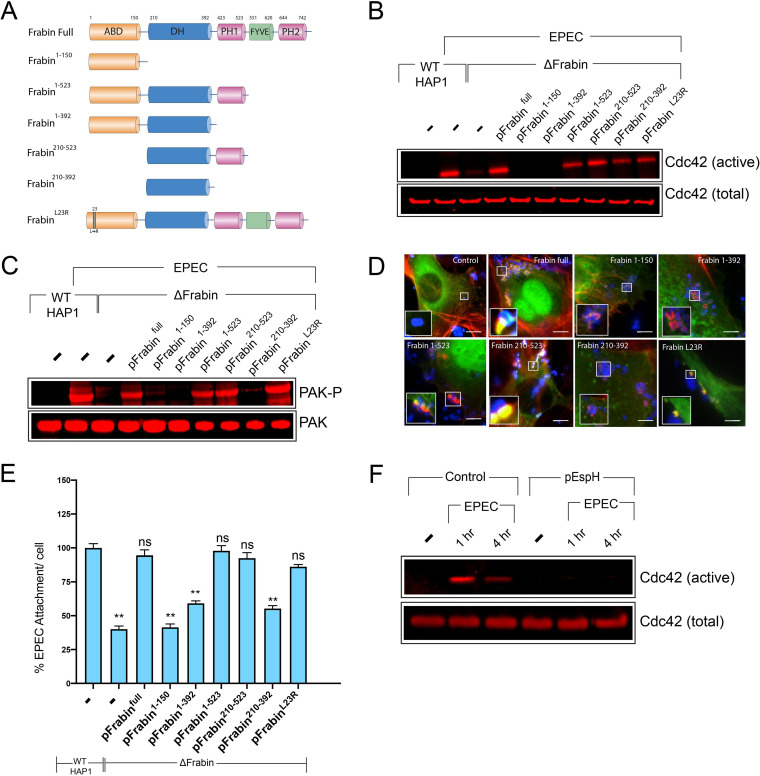
DH-PH1 domains of Frabin are sufficient for pedestal assembly. (A) Schematic showing the domain architecture of Frabin and the various derivative constructs used here. (B) Immunoblot of the level of Cdc42 isolated by GST-GBD beads (“active”) from lysates prepared from ΔFrabin HAP1 cells transfected with the indicated Frabin constructs and infected with WT EPEC. Also shown is the total level of Cdc42 in the lysates (“total”). (C) Immunoblot of the level of total (PAK) and active (phosphorylated on serine 144; PAK-P) PAK in ΔFrabin HAP1 cells transfected with the indicated Frabin constructs and infected with WT EPEC. (D) Fluorescence microscopy images of actin pedestals formed on ΔFrabin HAP1 cells either untransfected (control) or transfected with the indicated Frabin constructs. Actin (red) is stained with Texas Red-phalloidin, and bacteria (blue) are stained with anti-intimin antibody and frabin constructs (green). Scale bar, 10 μm. Insets magnify the highlighted area. (E) Quantification of the attachment of WT EPEC to ΔFrabin cells transfected with the indicated Frabin constructs relative to attachment of WT EPEC to WT HAP1 cells (there are typically 6 or 7 WT EPEC bacteria per WT HAP1 cell). Each bar represents the average of results from three separate experiments (100 to 200 cells for each replicate, 300 to 500 cells in total). Error bars indicate SD. **, *P* < 0.05; ns, not significant (by one-way analysis of variance [ANOVA] followed by a *post hoc* Dunnett comparison) relative to WT EPEC attachment. (F) Immunoblot of the level of Cdc42 isolated by GST-GBD beads (“active”) from lysates prepared from either untransfected (control) WT Hap1 cells, or those transfected with a plasmid expressing EspH (pEspH). Cells were either uninfected (−) or infected with WT EPEC for the indicated times. Also shown is the total level of Cdc42 in the lysates (“total”).

Frabin consists of multiple functional domains (Fig. 4A) (26). At the N terminus is an actin-binding domain (ABD), followed by a Dbl homology domain (DH) that possesses GEF activity, a lipid-binding pleckstrin homology domain (PH), a phosphatidylinositol-binding Fab1, YOTB, Vac1, and EEA1 domain (FYVE), and finally, a second PH domain. To determine which functional domains of Frabin are required for EPEC pedestal formation, we generated a series of derivative versions corresponding to various combinations of the individual domains (Fig. 4A) and expressed these versions in ΔFrabin cells prior to infection with EPEC. The ABD, FYVE, and both PH domains could all be deleted without impacting the EPEC-triggered Cdc42 activation (Fig. 4B, Fig. S4A). Perhaps unsurprisingly, the minimum required for GTPase activation was the DH, i.e., the site of GEF activity. However, the DH domain was inactive when fused to the ABD alone (Fig. 4B, Fig. S4A; pFrabin with amino acids 1 to 392 [pFrabin^1-392^]). This could be due to the ABD sequestering this construct to actin filaments, away from the plasma membrane where Rho GTPases are enriched. Indeed, Cdc42 activation was restored when a phospholipid-binding PH domain was also present (Fig. 4B, Fig. S4A; pFrabin^1-523^).

10.1128/mBio.01423-20.4 FIG S4 (A) Quantification of the level of Cdc42 isolated by GST-GBD beads (“active”) from lysates prepared from ΔFrabin HAP1 cells transfected with the indicated Frabin constructs and infected with WT EPEC. The levels are quantified from Western blot images shown in Fig. 4B. Each bar represents the average from three separate experiments; error bars represent SD. ***, *P* < 0.001; ns, not significant (one-way ANOVA followed by a *post hoc* Dunnett comparison) relative to control uninfected cells. (B)Quantification of the level of active (phosphorylated on serine 144; PAK-P) PAK in ΔFrabin HAP1 cells transfected with the indicated Frabin constructs and infected with WT EPEC. The levels are quantified from Western blot images shown in Fig. 4C and normalized by the level of total PAK. Each bar represents the average from three separate experiments; error bars represent SD. ***, *P* < 0.001; ns, not significant (one-way ANOVA followed by a *post hoc* Dunnett comparison) relative to control uninfected cells. (C) Quantification of the level of Cdc42 isolated by GST-GBD beads (“active”) from lysates prepared from either untransfected (control) WT Hap1 cells, or those transfected with a plasmid expressing EspH (pEspH). Cells were either uninfected (-) or infected with WT EPEC for the indicated times. The levels are quantified from Western blot images shown in Fig. 4F. Each bar represents the average from three separate experiments; error bars represent ± SD. ***, *P* < 0.001; *, *P* < 0.01; ns, not significant (one-way ANOVA followed by a *post hoc* Dunnett comparison) relative to control uninfected cells. Download FIG S4, TIF file, 0.3 MB. Copyright © 2020 Singh et al. 2020 Singh et al. This content is distributed under the terms of the Creative Commons Attribution 4.0 International license.

All of the Frabin constructs that were competent for EPEC-induced Cdc42 activation (Fig. 4B) were also able to promote PAK activation (Fig. 4C, Fig. S4B), with the exception of the DH domain alone (pFrabin^210-392^). As PAK activation could be triggered by the DH-PH domain combination (pFrabin^210-523^), this suggests that although the GEF domain alone is able to activate Cdc42 within the cell, the activation must occur at a specific subcellular location for PAK to be activated. Frabin constructs that were able to restore PAK activation in ΔFrabin cells were also able to restore pedestal formation (Fig. 4D) and bacterial attachment (Fig. 4E). Collectively, this demonstrates that Frabin is responsible for EPEC-induced Cdc42 activation, via its DH domain, and that the presence of the PH domain is required to ensure that this Cdc42 activation occurs in the correct location within the cell to allow PAK activation and consequent bacterial attachment to the host cell.

Presumably, once pedestals have been produced, the Frabin pathway may be switched off. Consistent with this, the levels of active Cdc42 are approximately 50% lower at 4 h compared to 1 h postinfection (Fig. S4C). Intriguingly, EPEC encodes an effector, EspH, that can inhibit certain mammalian GEFs (27, 28). Transfection of HAP1 cells with a plasmid expressing EspH abolished EPEC-induced Cdc42 activation at all time points (Fig. 4F, Fig. S4C), suggesting it could play a role in downregulating the Frabin pathway following pedestal production.

### PIP2 and Arf6 are required to localize Frabin.

**FIG 5:**
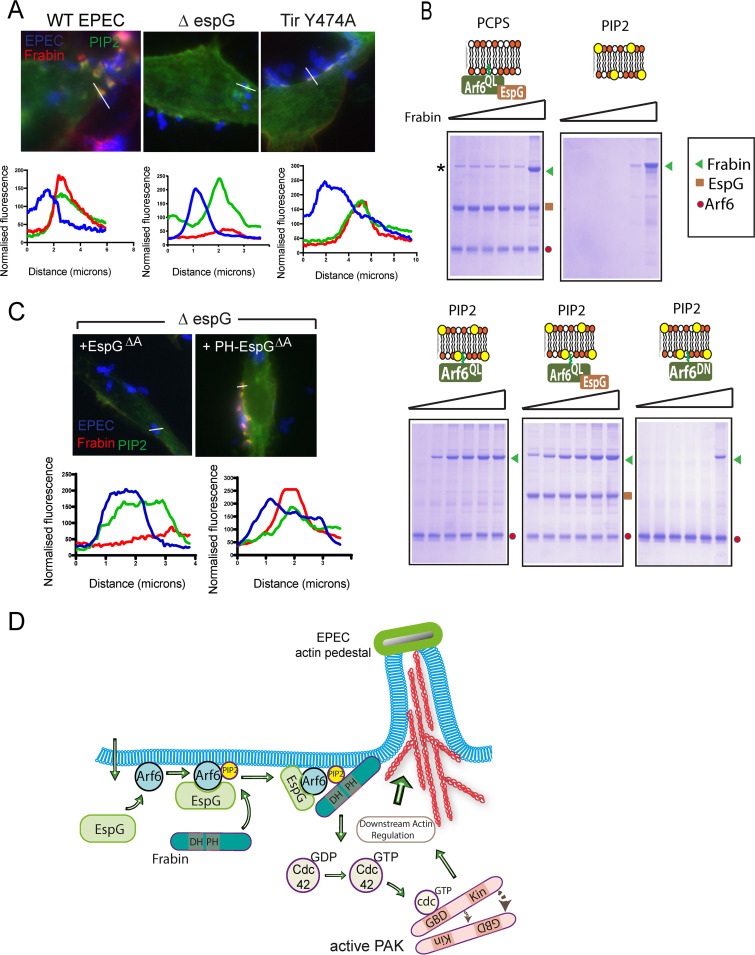
Factors determining Frabin recruitment to the pedestal. (A, top) Fluorescence microscopy images of actin pedestals formed on WT HAP1 cells expressing dsRed-Frabin (red) and a PIP2 marker (the PH domain from phospholipase C δ1 fused to enhanced green fluorescent protein [EGFP]; green) by WT EPEC, Δ*espG* EPEC, or Δ*tir* EPEC transformed with a plasmid encoding Tir^Y474A^ (blue). (Bottom) Pixel (fluorescence) intensity plots, measured along the indicated lines in micrographs. (B) SDS-PAGE analysis and Coomassie blue staining of proteins present on PC:PI or PC:PI:PIP2 (PIP2) bilayers anchored with the proteins indicated in the cartoons (Arf6^QL^ alone or with coanchored EspG), following incubation with increasing concentrations of purified, recombinant Frabin. Mobility of Frabin, EspG, and Arf6 are indicated on the right-hand side of each panel (green triangle, brown square, and red circle, respectively). The asterisk indicates a minor contaminant in the recombinant EspG preparation that interacts with PC:PI bilayers and migrates slightly above the position of Frabin. (C, top) Fluorescence microscopy images of actin pedestals formed by WT EPEC (blue) on WT HAP1 cells transfected with dsRed-Frabin (red), PIP2 marker (green), and either EspG^ΔA^ or PH-EspG^ΔA^, as indicated. (Bottom) Pixel (fluorescence) intensity plots, measured along the indicated lines in micrographs. (D) Model of Frabin’s role in pedestal assembly. See Discussion for a full description.

The first Frabin PH domain, required for EPEC to able to activate PAK, is known to bind to phosphatidylinositol 4,5-bisphosphate (PIP2), though its binding specificity remains unclear (29). PIP2 was present in the lipid bilayer-coated microspheres used to identify Frabin as a target for EspG (Fig. 2), and it is known to be enriched at sites of EPEC pedestal formation (23). Consistent with the role of the PH domain, Frabin colocalized with PIP2, immediately below sites of EPEC attachment to cultured Hap1 cells (Fig. 5A). In the absence of EspG, although PIP2 was present at sites of EPEC attachment, Frabin was not recruited (Fig. 5A). When Hap1 cells were infected with EPEC that expresses Tir^Y474A^, a strain which attaches but is unable to generate actin pedestals, both PIP2 and frabin were still recruited to sites of bacterial adhesion (Fig. 5A), suggesting that actin polymerization is not required for Frabin recruitment by EspG.

As EspG is required for Frabin association with sites of EPEC attachment, we sought to determine whether EspG directly interacts with Frabin by reconstituting Frabin recruitment to lipid bilayers using purified recombinant proteins. Microspheres coated with a bilayer composed of phosphatidylcholine and phosphatidylinositol (PC:PI), and anchored with EspG and constitutively active Arf6^QL^ (required for EspG membrane association) showed minimal recruitment of Frabin, with bound Frabin visible only when present at high concentrations (Fig. 5B). Recombinant Frabin could be efficiently recruited however when EspG and Arf6^QL^ were anchored to bilayers also containing PIP2, but not to control PIP2 microspheres in the absence of Arf6^QL^ and EspG. Surprisingly, Frabin was also recruited, though less efficiently, to control Arf6^QL^ PIP2 microspheres, though not to inactive, GDP-bound Arf6 (Fig. 5B).

It is therefore possible that active Arf6 and PIP2 are responsible for Frabin enrichment at sites of EPEC attachment and that, as EspG is known to prevent cellular Arf GAPs from inactivating Arfs (30), the role of EspG is to enhance the local level of active Arf. Alternatively, it is possible that Frabin is able to directly interact with either Arf6 or EspG. As Arf6 is required for anchoring EspG to lipid bilayers in our *in vitro* reconstitution system, we returned to cell infection to further test the role of EspG. When Δ*espG* EPEC was used to infect Hap1 cells transfected with EspG^ΔA^ (a derivative incapable of binding Arfs and therefore unable to localize to the host cell membrane), Frabin was not significantly enriched at sites of bacterial attachment (Fig. 5C). However, when EspG^ΔA^ is artificially targeted to the membrane by fusing to a lipid-binding PH domain (PH-EspG^ΔA^), Frabin was efficiently recruited to sites of EPEC attachment. This suggests that EspG can enhance recruitment of Frabin without interacting with Arf6, and therefore does not act to simply protect active Arf6 from inactivation.

## DISCUSSION

Collectively, the results presented here allow us to propose a model by which EPEC ensures that target cells contain the high levels of localized active Rho GTPases necessary for subversion of PAK signaling (Fig. 5D). Upon injection by the T3SS, EspG forms a complex with active, GTP-bound Arf6 at the plasma membrane. EspG binding sterically blocks cellular Arf GAPs from binding and inactivating Arf6. The initial attachment of EPEC leads to the accumulation of PIP2 directly beneath the adherent bacteria. The coincident PIP2 and EspG-Arf6 lead to enrichment of Frabin, triggering local activation of Cdc42. This in turn leads to activation of PAK, which is hijacked by EspG-Arf6 to promote the actin rearrangements necessary for pedestal formation and intimate EPEC attachment to the host cell.

Active Rho GTPases are a requirement for exploitation of PAK by EPEC (10). Rho GTPases are master regulators of multiple signaling pathways within eukaryotic cells, and consequently, their activation status is spatially and temporally dynamic (11). If EPEC encounters a cell in which these pathways are already active, which in cell culture may depend on specific cell line and growth conditions, the Frabin pathway may not be as important for pedestal generation. This possibly explains why previous studies have failed to identify a role for Rho GTPases in pedestal formation (31). However, when needed, by promoting Frabin activation directly beneath adherent bacteria, EPEC ensures that local PAK will always be in the GTPase-activated state required for EspG hijacking.

Little is known about the function of Frabin in healthy cells. Various mutations in the Frabin gene have been linked to the hereditary neuropathy Charcot-Marie-Tooth disease (32), and Frabin may play a role in myelin homeostasis in the peripheral nervous system (33). Frabin overexpression may also play a role in certain cancers (34). The presence of three potential lipid-binding domains (two PH domains and a FYVE domain), and an actin-binding domain suggest it is able to localize to various sites within the cell to perform multiple functions (29), and indeed, Frabin has been seen to localize to several actin-associated membrane structures (35). Here we show that the role of Frabin in EPEC pedestal formation requires only the DH (GEF) domain and the first PH domain. PH domains are found in many proteins and are known to target cellular membranes by binding to phosphoinositide lipids and proteins (36). Indeed, some PH domains are able to bind to both lipids and small GTPases, and they have been reported to act as coincidence detectors (37). For example, Fapp1 (four-phosphate adaptor protein 1) is targeted to membranes by its PH domain, which detects coincident Arf1 and phosphatidylinositol 4-phosphate (38).

Frabin enrichment at sites of EPEC attachment also seems to require two coincident signals. The first of these, PIP2, is known to be enriched directly beneath the adherent bacteria (23). Indeed, disruption of this PIP2 accumulation was shown previously to severely impair EPEC attachment and actin pedestal formation (23), which the results presented here suggest may be due at least in part to impaired Frabin localization. The second required signal for Frabin recruitment could be supplied by constitutively active Arf6^QL^ *in vitro* using purified proteins (Fig. 5B), but during cell infection EspG was necessary (Fig. 5A). As EspG is known to interact with Arfs and protect them from inactivation (30), the requirement for EspG could be due to it enhancing the level of active Arf present at the site of bacterial attachment. However, as a mutant of EspG unable to interact with Arfs could efficiently recruit Frabin when artificially targeted to the membrane, it is possible that EspG can directly interact with Frabin. Nevertheless, during infection with WT EPEC, EspG localizes to bacterial attachment sites by binding Arf6, and thus both potential Frabin interactors are present together and may well cooperate. It should also be noted that recruitment to membranes does not necessarily correlate with activation of Frabin’s GEF activity (i.e., the DH domain), and the requirement of EspG for Cdc42 activation may be indicative of a direct role in activating Frabin.

The molecular details of the complex interactions regulating this pathway will require further study, but we have identified Frabin as a key component of the signaling complex assembled by EspG to ensure that conditions within the target cell are permissive for EPEC to form actin pedestals. This is the first report of Frabin being involved in bacterial pathogenesis. Previously, Frabin has been shown to be required for the entry of the intracellular parasite Cryptosporidium parvum into epithelial cells by promoting Cdc42 activation and consequent downstream actin rearrangements (39). Though the molecular details of this pathway remain unknown, as seen here with EPEC the recruitment of Frabin by C. parvum required the generation of phosphorylated phosphoinositide lipids in the target cell membrane. Epstein-Barr virus also subverts Frabin, leading to Cdc42 activation and enhanced motility of virus-infected cells (40). Rho GTPases such as Cdc42 are ubiquitous targets for pathogenic bacteria due to their central role in myriad cell signaling pathways (41). Likewise, many bacteria manipulate phosphoinositide lipids in target cell membranes (42). It is thus intriguing to speculate that Frabin may be an important component of the signaling networks subverted by other pathogens to drive the cytoskeletal changes underlying pathogenesis.

## MATERIALS AND METHODS

### Bacterial strains.

EPEC E2348/69, isogenic mutant EPEC Δ*espG1*/Δ*espG2* (Feng Shao), and EPEC Δ*map* (Gad Frankel) were generous gifts.

### Plasmids.

The plasmids pTrcEspG, pTrcEspGΔR, pTrcEspGΔP, pTrcEspGΔAP, pTrcEspGΔA, pCDNA-HA-EspGΔA, and pCDNA-HA-PH-EspGΔA were described previously (10, 43). Plasmids pET20b-Arf6, pET15b-espG, pET20b-Frabin, pCDNAnHA-Frabin^WT^, pCDNAnHA-Frabin^1-150^, pCDNAnHA-Frabin^1-392^, pCDNAnHA-Frabin^1-523^, pCDNAnHA-Frabin^210-523^, pCDNAnHA-Frabin^210-392^, and pCDNAnHA-Frabin^L23R^ were generated using Gateway methodology (Invitrogen).

### Antibodies.

Antibodies were supplied by the following companies: Cell Signaling Technology (phospho-PAK1 (Ser144)/PAK2 (Ser141), catalog no. 2606; PAK1, catalog no. 2602); Santa Cruz Biotechnology (Frabin, Sc-136333); Abcam (Rac1, ab33186; Arf6, ab81650; tubulin, ab7291); Sigma (actin, catalog no. A2066); BD Bioscience (Cdc42, catalog no. 610929); Qiagen (His tag, catalog no. 34660). Rabbit anti-intimin was raised against full-length recombinant initimin by Diagnostics Scotland.

### Mammalian cell culture and transfection.

The WT Hap1 cells (C631) and verified knockout lines ΔArf6 (HZGHC003403c006), ΔFrabin (HZGHC004680c010), and ΔPak1 (HZGHC000160c012) were purchased from Horizon Discovery. Cells were maintained in Iscove modified Dulbecco medium (IMDM) supplemented with 10% fetal bovine serum (FBS) and 100 U/ml penicillin-streptomycin. Where indicated, cells were preincubated for 60 min prior to bacterial infection with 10 μM concentration of the inhibitor EHT1864 (Merck) or 25 μM PAK inhibitor (FRAX486, Tocris).

Where indicated, plasmids were introduced into mammalian cells using the Neon transfection system (Invitrogen) according to the manufacturer’s instructions.

Flexitube siRNAs (Qiagen) used to knockdown Frabin (Hs_FGD4_1 NM_139241 and Hs_FGD4_2 NM_139241) were transfected using Oligofectamine (Life Technologies) following the manufacturer’s instructions.

### Attachment assay and pedestal quantification.

Cells were infected as described previously (44). For pedestal quantification, cells were washed three times with phosphate-buffered saline (PBS), fixed, and stained with Alexa Fluor 488 phalloidin (Lifetech) to visualize actin and an anti-intimin antibody to visualize the bacteria. The numbers of actin pedestals per cell were then counted using fluorescence microscopy. For adhesion assays, after infection, cells were washed twice with PBS and then twice briefly with 200 mM glycine (pH 2), followed by a further two washes with PBS. Cells were fixed and stained as described above, and the number of bacteria still adherent was counted using microscopy.

The pedestal length was determined by drawing a line beneath the attached bacteria and measuring the length using ImageJ (FIJI) tool. A minimum of 150 attached bacteria were counted per condition to estimate the average pedestal length for a given condition. Pixel (fluorescence) intensity plots were generated using the “plot profile” tool of ImageJ (FIJI), and the lines were drawn through pedestal or pedestal-forming regions.

### GST-PBD GTPase activation assay.

Active GTPases were detected as described previously (45). Briefly, indicated cells were washed twice in ice-cold PBS and then lysed in radioimmunoprecipitation assay (RIPA) buffer (Sigma-Aldrich) supplemented with a cocktail of protease inhibitors. Cell lysates were subjected to centrifugation (13,000 × *g*, 15 min, 4°C) to remove insoluble material. Equivalent amounts of cleared lysates were then incubated with GST-PBD bound to glutathione-Sepharose resin (GE Healthcare) at 4°C for 30 min. Resin was packed onto a column, extensively washed, and then bound Cdc42 and Rac1 were eluted using SDS-urea. The samples were then analyzed using SDS-PAGE and Western blotting.

### *In vitro* pulldown assays.

Pulldowns were performed as previously described (46). Briefly, silica microspheres (Bangs Laboratories) were coated with a bilayer composed of either phosphatidylcholine (PC) and phosphatidylserine (PS), at a molar ratio of 80:20 PC:PS, or phosphatidylcholine (PC), phosphatidylinositol (PI) and phosphatidylinositol 4,5-bisphosphate (PIP2), at a 48:48:4 molar ratio of PC:PI:PIP2. All lipids were purchased from Avanti Polar Lipids. Indicated proteins were anchored to these bilayers, prior to incubation in cell-free porcine brain extract. Following incubation for 15 min, bilayers were washed extensively and associated proteins were analyzed by SDS-PAGE. To identify recruited proteins, beads were washed with 50 mM ammonium bicarbonate (pH 8.5) supplemented with 0.5 mM dithiothreitol (DTT) and then were incubated with trypsin. The digested proteins were identified by electrospray ionization liquid chromatography mass spectrometry (MS) (Mass Spectrometry Service, Cambridge Centre for Proteomics, University of Cambridge, Cambridge, UK). The MS fragmentation data were used to search the National Center for Biotechnology Information (NCBI) database using the MASCOT search engine (www.matrixscience.com).

### PAK activation assays.

PAK activation was measured as previously described (10). Briefly, cells were cultured in 10-cm dishes and starved in serum-free IMDM overnight. The following day, cells were infected (90 min) or treated with appropriate drugs, as indicated, and then washed twice with PBS before being detached using a cell scraper and resuspended in SDS-urea. Samples were analyzed by SDS-PAGE and immunoblotting using appropriate antibodies. Immunoblots are representative of at least three separate repeats. Bands were visualized using a LI-COR Odyssey Fc imaging system, and band intensities were quantified using the LI-COR Image Studio software.
