# Supplementary figures and images for: EPEC Recruits a Cdc42-Specific GEF, Frabin, To Facilitate PAK Activation and Host Cell Colonization
Source: mBio. 2020 Nov 3;11(6):e01423-20. doi: 10.1128/mBio.01423-20 (PMC7642674; doi:10.1128/mBio.01423-20)

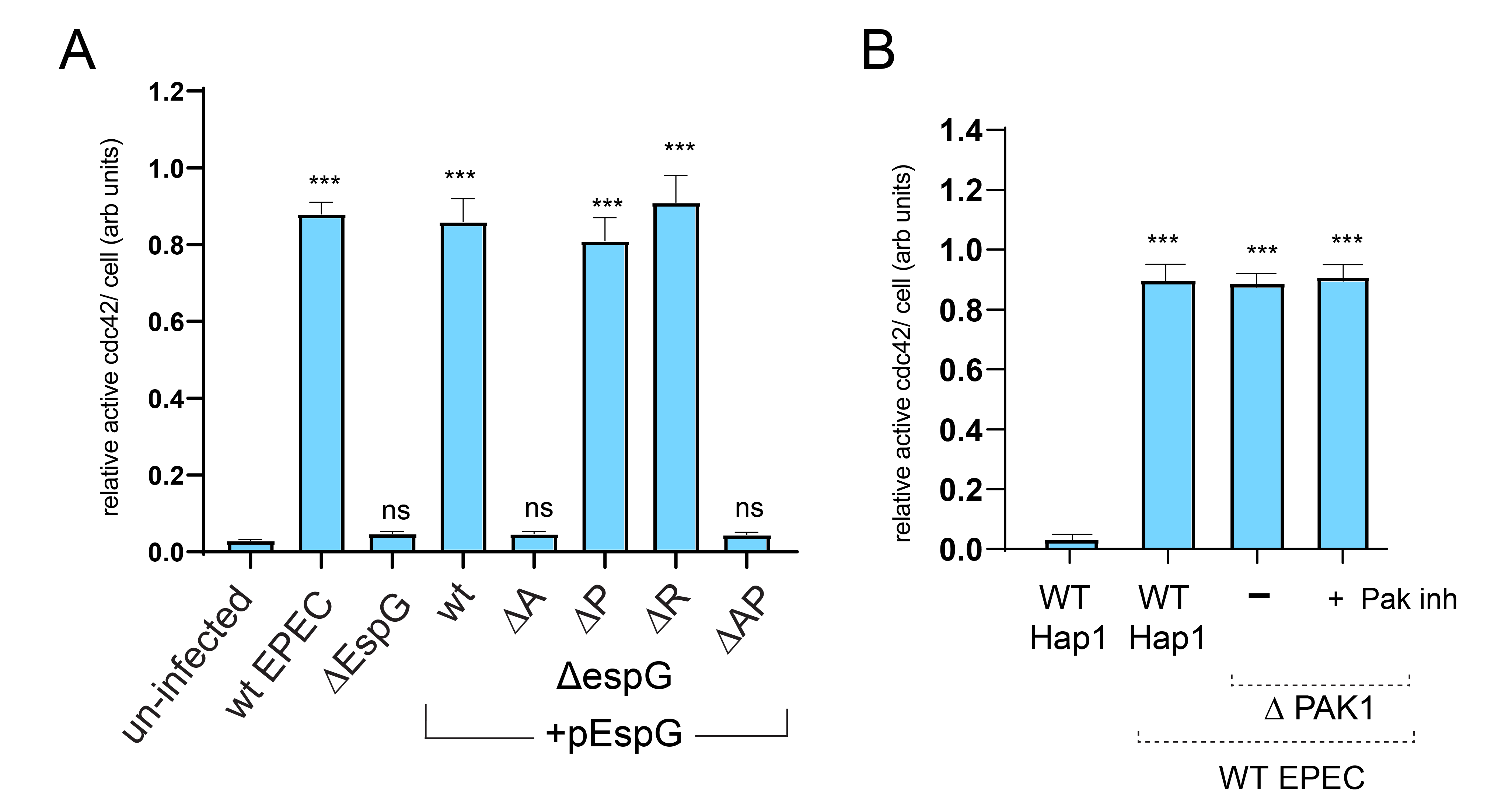

Supplement: FIG S2 [file mBio.01423-20-sf002.tif]

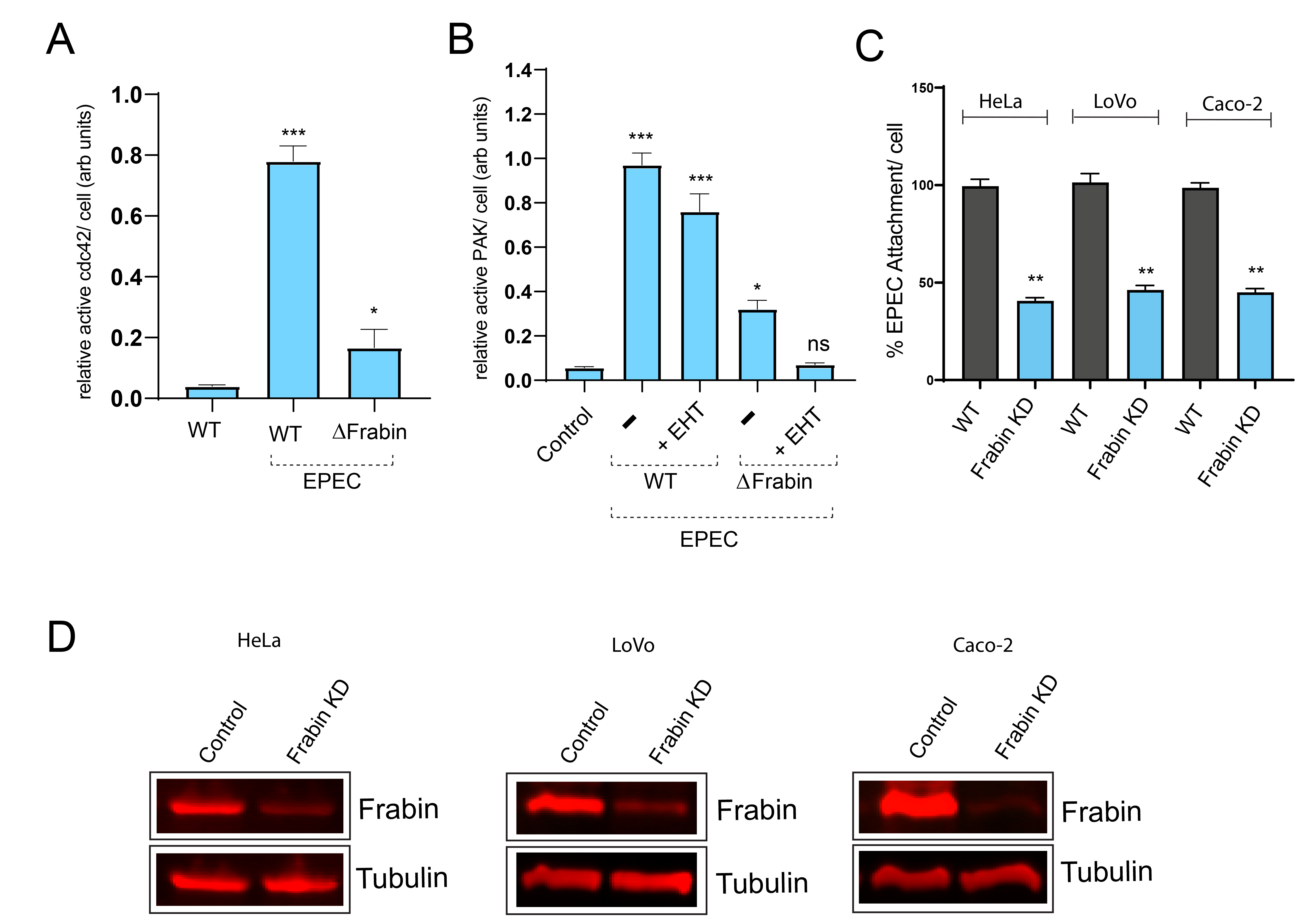

Supplement: FIG S3 [file mBio.01423-20-sf003.tif]

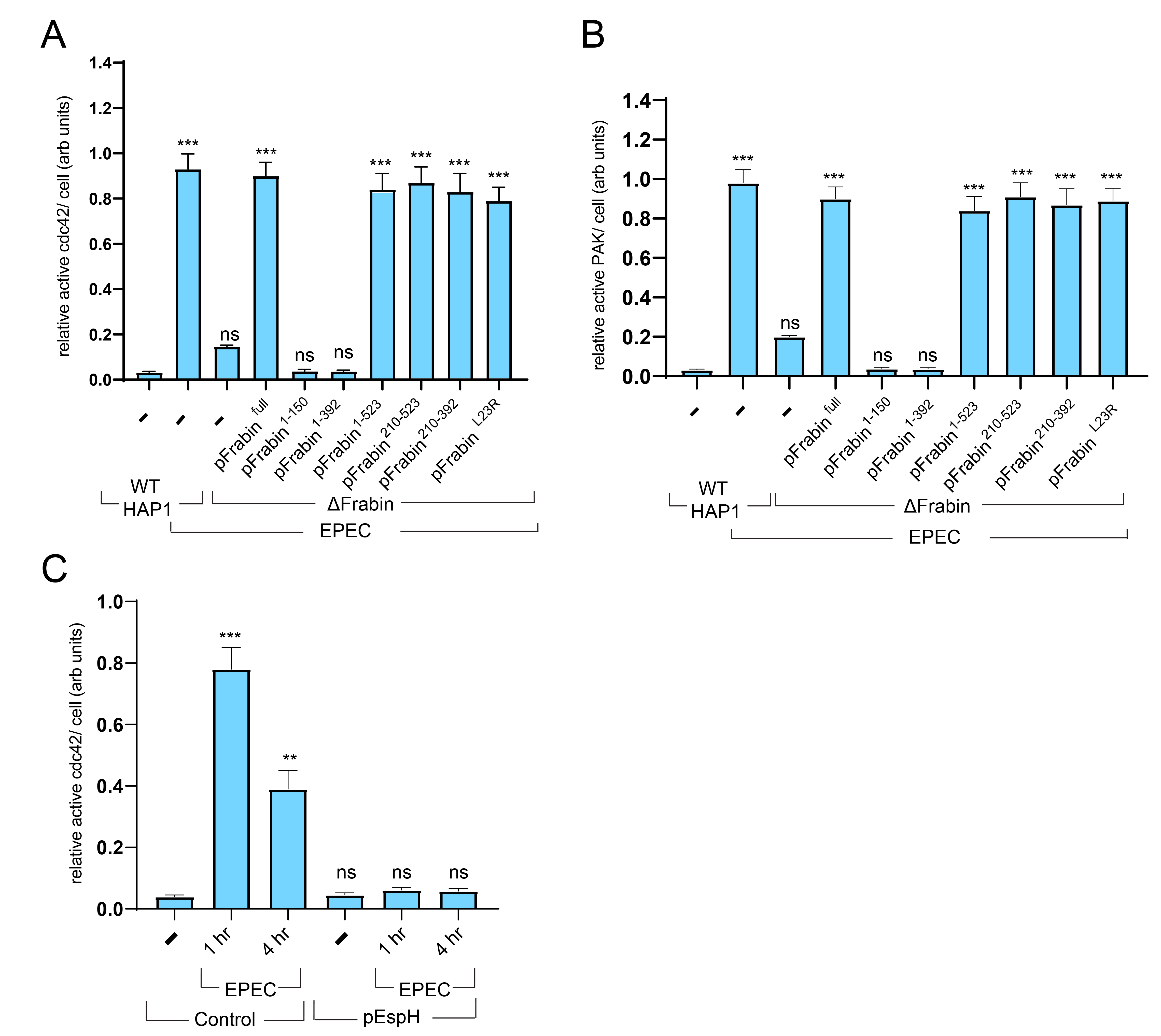

Supplement: FIG S4 [file mBio.01423-20-sf004.tif]
